# A Perspective on the Use of Hydroxyapatites to Improve the Dissolution Behavior of Poorly Water-Soluble Piretanide

**DOI:** 10.3390/pharmaceutics16111450

**Published:** 2024-11-13

**Authors:** Valeria Friuli, Claudia Loi, Giovanna Bruni, Lauretta Maggi, Marcella Bini

**Affiliations:** 1 Department of Drug Sciences, University of Pavia, viale Taramelli 12, 27100 Pavia, Italy; valeria.friuli@unipv.it (V.F.); lauretta.maggi@unipv.it (L.M.); 2 Department of Chemistry, University of Pavia, viale Taramelli 16, 27100 Pavia, Italy; claudia.loi01@universitadipavia.it (C.L.); giovanna.bruni@unipv.it (G.B.); 3 CSGI—Department of Chemistry, University of Pavia, viale Taramelli 16, 27100 Pavia, Italy; 4 National Reference Centre for Electrochemical Energy Storage (GISEL)—INSTM, Via G. Giusti 9, 50121 Firenze, Italy

**Keywords:** hydroxyapatite, piretanide, doping, drug delivery, dissolution rate, wettability

## Abstract

Background/Objectives: Interest in drug delivery systems (DDS) based on inorganic substrates has increased in parallel with the increase in the number of poorly water-soluble drugs. Hydroxyapatite is one of the ideal matrices for DDS due to its biocompatibility, low cost, and ease of preparation. Methods: We propose two doped hydroxyapatites, one with Ba on Ca sites another with Si on P sites, with the aim of improving the dissolution rate of piretanide, a diuretic, poorly water-soluble drug. The hybrids were characterized by different physical–chemical techniques, and their formation was demonstrated by infrared spectroscopy, thermal analysis, and electron microanalysis, as well as by comparing the results with those obtained on physical mixtures of HAPs and properly prepared piretanide. Results: Both the hybrids improved the piretanide dissolution rate compared with the physical mixtures and the drug alone. The dose was completely solubilized from the Si-doped hybrid in about 5 min in the three fluids considered. This remarkable improvement can be explained by an increase in the wettability and solubility of the drug loaded in the drug-carrier systems. Conclusions: Different experimental techniques, in particular spectroscopy and electronic microanalysis, proved the successful loading of piretanide onto doped HAP. Pharmaceutical measurements demonstrated rapid drug release in different fluids simulating gastrointestinal conditions after oral administration. These hybrid systems could be a very promising platform for drug delivery.

## 1. Introduction

In recent years, hybrid systems, comprising host inorganic matrices and drug guests, have represented a promising strategy for the pharmaceutical industry to develop new drug alternatives [1,2]. In particular, the hydroxyapatite (HAP) Ca_10_(PO_4_)_6_(OH)_2_, which is the main constituent of biological tissues, such as bones and teeth, is considered an appealing matrix due to its many advantages, the main one being high biocompatibility [3,4,5]. Other intriguing features of HAP are represented by its biodegradability, osteogenesis, osteoconductivity, and bioactivity; in addition, HAP can form direct bonds with living tissues [6,7]. Tissue engineering, nanomedicine, industrial catalysis, and orthopedic implant coating [8,9] represent some of the application fields of HAP powders with different external forms. No toxicity toward other organs was observed when drug delivery was performed using hydroxyapatite, and hybrids can avoid repeated drug dosing. HAP can also easily bond with different molecules by adsorption and its use has been proposed for the release of many different molecules like antibiotics, anti-inflammatories, hormones, and anticancer drugs [10,11,12]. It is very important to obtain HAP particles with customized properties that can find applications in both bone-tissue engineering and drug delivery fields. It was demonstrated that bone regeneration and growth could be improved by proper tailoring of the physical–chemical, morphological, and biological properties of HAP. In addition, it has been observed that the morphology and possible porous structure of HAP nanoparticles have a direct effect on the amount of loading and release profiles of a specific drug [3]. The structure of HAP can host many kinds of ions and, in fact, the doping/substitution of HAP with different elements (e.g., Mg, Si, Sr, Eu, Zn, Ce) has been suggested to improve drug loading compared with HAP alone [13,14]. In addition, substituents, which can be also intended as micronutrients for the body, can produce additional effects.

Piretanide (4-phenoxy-3-(pyrrolidine-1-yl)-5-sulfamoyl benzoic acid) is a loop diuretic useful in cases of heart failure, hypertension, and edematous states that can occur in patients with renal and hepatic diseases [15]. Piretanide has a structure that is similar to that of furosemide and bumetanide, but it has the advantage of a low therapeutic dose (6 mg instead of 40 mg). Piretanide can be classified as a poorly soluble drug [16,17] (pKa = 3.85) [18] (its % ionization as a function of the pH is reported in Figure S1); in this regard, the improvement of its release rate represents a challenge. As far as we know, there has been only one paper reporting on the use of piretanide–LDH (layered double hydroxide) hybrids to increase the solubility and dissolution rate [19].

We aimed to prepare doped hydroxyapatite-based drug delivery systems to improve the pharmaceutical features of piretanide. We chose two substituent ions not often reported in the literature, substituting calcium or phosphorous. In particular, we used barium ions on calcium sites [20] and silicon as a substitute for phosphorous. It is reported that silicon is an important element for the structural integrity of nails, hair, and skin; for bone mineralization and bone health; and for reducing metal accumulation in Alzheimer’s disease and the risk of atherosclerosis [14,21].

A combined use of different techniques was useful to characterize the obtained hybrids, whose formation was demonstrated by scanning electron microscopy (SEM) coupled with energy-dispersive X-ray spectroscopy (EDS) microanalysis. X-ray powder diffraction (XRPD), differential scanning calorimetry (DSC), and infrared spectroscopy (FT-IR) were particularly useful in proving the presence of the drug. The measurement of contact angles and solubility and the in vitro dissolution tests, performed in different media simulating the gastric environment in fed and fasted conditions, demonstrated that the hybrids were effective in improving piretanide’s wettability and dissolution rate.

## 2. Materials and Methods

### 2.1. Materials

**Scheme 1:**
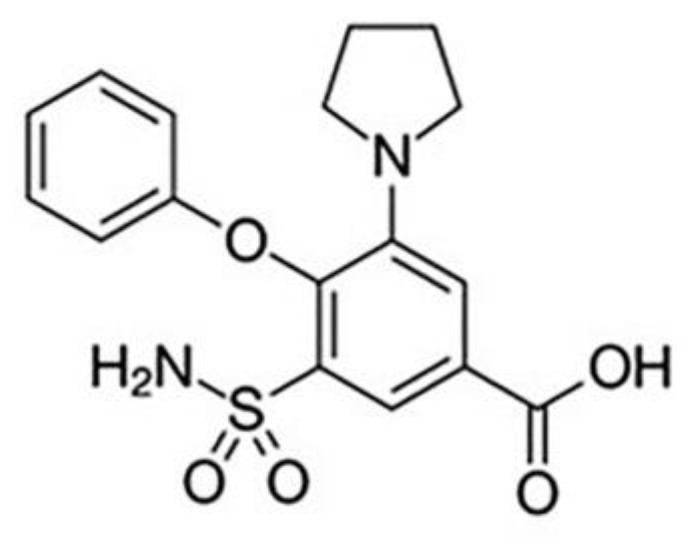
Molecular structure of piretanide.

Calcium nitrate tetrahydrate Ca(NO_3_)_2_ 4 H_2_O (puriss.), barium nitrate Ba(NO_3_)_2_ (99+%), tetraethyl ortho silicate (TEOS), potassium phosphate monobasic KH_2_PO_4_, dibasic ammonium phosphate (NH_4_)_2_HPO_4_ (≥99%), ammonium hydroxide, sodium hydroxide, and HCl 36% were purchased from Sigma-Adrich, St. Louis, MO, USA. Piretanide (Pir) (Scheme 1) was generously donated by Olon, Casaletto Lodigiano (LO), Italy.

### 2.2. Syntheses

#### 2.2.1. Synthesis of Hydroxyapatites

Ba-doped hydroxyapatite (Ca_9.5_Ba_0.5_(PO_4_)_6_(OH)_2_) was synthesized by means of the co-precipitation method. Ca(NO_3_)_2_ 4 H_2_O and Ba(NO_3_)_2_ (9.5:0.5 molar ratio) were dissolved in 30 mL of distilled water (solution A), while (NH_4_)_2_HPO_4_ was dissolved in another 30 mL of water (solution B). NH_4_OH was added until pH~10 was reached in both solutions. After stirring until the reagents had dissolved, solution B was poured drop by drop into solution A, after which the pH was checked and, if necessary, adjusted to about 10 with ammonium hydroxide. After 30 min of stirring, the temperature was raised to 80 °C and maintained for 1 h. After cooling to room temperature, the solution was centrifuged, and the collected powder was washed three times with deionized water and then transferred to an oven at 100 °C for 22 h. This sample is named HAP-Ba.

Si-doped hydroxyapatite (Ca_10_(PO_4_)_5.6_(SiO_4_)_0.4_(OH)_2_ was also synthesized by co-precipitation. The source of silicon was tetra ethyl orthosilicate (TEOS). Ca(NO_3_)_2_ 4 H_2_O was dissolved in 30 mL of distilled water, the temperature was increased to 80 °C, and then the required amount of TEOS was added (solution A). (NH_4_)_2_HPO_4_ was dissolved in another 30 mL of water (solution B). NH_4_OH was added until pH~10 was reached in both solutions. Solution B was added, as for Ba-doped HAP, to solution A. The resulting solution was maintained at 80 °C for 3 h, then cooled to room temperature and aged for 12 h. After that, the precipitated powder was centrifuged, washed three times with water, and then dried in a muffle furnace for 24 h at 80 °C. This sample is named HAP-Si.

#### 2.2.2. Synthesis of HAPs@Piretanide Hybrids

A mixture of ethanol:water (3:1 v:v ratio) (drug concentration 12.5 mg/mL; 8 mL overall) was used to dissolve piretanide. An amount of 150 mg of HAP-Ba or HAP-Si was then added to the drug solution, and the obtained dispersions were stirred at ambient temperature for 24 h. Finally, the powders were centrifuged, dried at 60 °C overnight, and stored in a desiccator for subsequent use. In the following sections, these samples will be known as HAP-Ba-Pir and HAP-Si-Pir. 

### 2.3. Techniques

#### 2.3.1. Physical–Chemical Measurements

X-ray powder diffraction measurements were performed using a Bruker D5005 diffractometer (Bruker BioSpin; Fällanden, Switzerland) equipped with Cu Kα radiation, a graphite monochromator, and a scintillation detector. The patterns were collected in the air with a step size of 0.03° and counting time of 2 s per step in the angular range of 10–70° (hybrids and HAPs), 3–40° (Pir) and 5–40° (physical mixtures) using a low-background silicon sample holder. 

A Nicolet FT-IR iS20 spectrometer (Nicolet, Madison, WI, USA) equipped with an ATR (attenuated total reflectance) sampling accessory (Smart iTR with diamond plate) was used to obtain the FT-IR spectra by co-adding 32 scans in the 4000–600 cm^−1^ range at a 4 cm^−1^ resolution.

Differential scanning calorimetry measurements were performed by a DSC Q2000 apparatus interfaced with a TA 5000 data station (TA Instruments, New Castle, DE, USA). The standard chosen for the instrument calibration was ultrapure indium (m.p. = 156.6 °C; Δ*H* = 28.54 J g^−1^). The calorimetric measurements were performed at up to 250 °C (heating rate 10 °C min^−1^) on samples amounting to about 3 mg in open standard aluminium pans under nitrogen flow (45 mL·min^−1^).

A Zeiss Evo MA10 (Carl Zeiss, Oberkochen, Germany) microscope coupled with the EDS detector for microanalysis (X-max 50 mm, Oxford Instruments, Abingdon, UK) was used to collect SEM images and microanalysis spectra. The samples for SEM analysis were sputtered with a thin layer of gold and analyzed at the acceleration voltage of an electron beam of 20 kV. The EDS data were obtained on pristine samples (not sputtered). 

#### 2.3.2. Pharmaceutical Characterization

The samples were previously sieved through a 53 µm sieve (ASTM Endecotts LTD, London, UK). Drug loading was determined under conditions in which piretanide is most soluble in a volume sufficient to ensure “sink conditions”, i.e., in deionized water, with UV-Vis spectroscopy (Lambda 25 UV spectrophotometer; Perkin-Elmer, Monza, Italy) at a wavelength of 274 nm after performing a calibration curve. The UV-Vis spectrum of HAP had been previously acquired to verify that there was no overlap in carrier absorption with respect to the drug at the wavelength used. Once the drug loading was determined, the corresponding physical mixtures were prepared in the same ratio (pmHAP-Si-Pir and pmHAP-Ba-Pir) using a Turbula mixer (Bachofen, Basel CH, Switzerland).

The solubility of the drug and hybrids was determined in triplicate using the shake-flask method in deionized water and HCl 0.1N pH = 1.0 (which simulates the fasted gastric environment) [22] at 21 °C. An excess sample was poured into volumetric flasks and left under magnetic stirring at 100 rpm. The supernatant was filtered (0.22 μm; Millipore, Merk KGgA, Darmstadt D) and diluted, and the concentration was determined by UV absorbance at different time intervals until equilibrium was reached.

The dissolution tests (triplicate) were performed using USP Apparatus 2 (paddle) (Erweka DT-D6; Erweka, Dusseldorf, D) at 37 °C and a 50 rpm stirring rate. The vessels were filled with 900 mL of deionized water or two other fluids to simulate oral administration: HCl 0.1N pH = 1.0, which simulates the gastric environment in the fasted condition, and phosphate buffer pH = 4.5 [22], which mimics the fed gastric condition. The dissolution tests were carried out in these two fluids because, in vivo, piretanide first encounters these environments. Moreover, the drug has a pH-dependent solubility, i.e., it is less soluble in acidic and neutral fluids and more soluble at a basic pH. Thus, these conditions are the most selective for highlighting differences in the behavior of the hybrids. The drug concentrations were measured by UV-Vis absorbance (Lambda 25; Perkin-Elmer, Monza, Italy) on filtered portions of the dissolution medium; the dose was always 6 mg. The data were processed using suitable software (Winlab V6 software; Perkin-Elmer, Monza, Italy) to obtain the dissolution profiles. The f_2_ test evaluated the similarities or differences between the dissolution curves obtained [23].

The contact angles were measured on the different powder samples. Some images were taken of a 9 µL drop (of the same fluids used for the dissolution test) deposited on the surface of the samples during the time, from t = 0 up to 300 s, by the contact-angle meter DMe-211Plus (NTG Nuova Tecnogalenica, Cernusco, Italy). The software supplied by the manufacturer processed the results in terms of the angle between the liquid–air and liquid–solid interfaces (three replicates).

## 3. Results

### 3.1. Physical–Chemical Characterization

**Figure 1:**
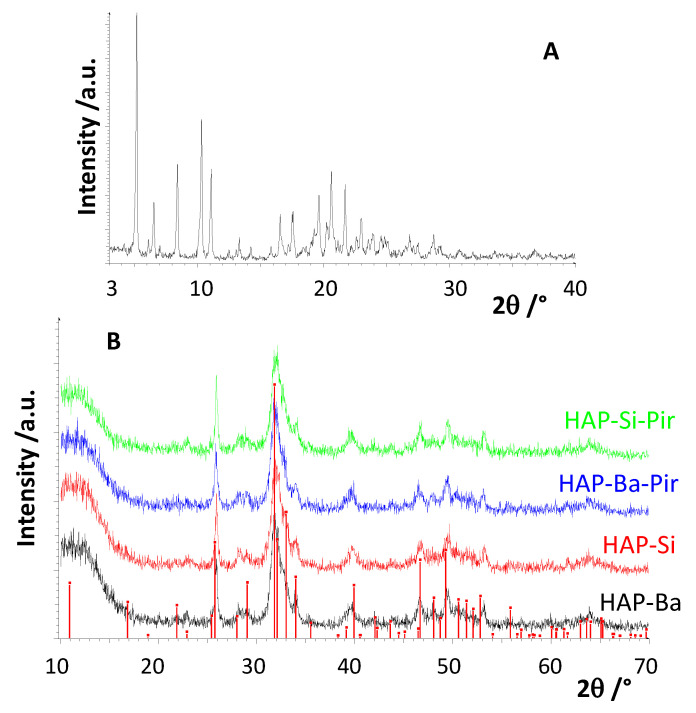
XRPD patterns of (**A**) piretanide and (**B**) the HAPs and hybrids, respectively. The red bars refer to the expected peak positions of hydroxyapatite (Card N. 73-1731).

The XRPD analysis was the first attempt to characterize the materials to verify the formation of the desired products. In our case, it was applied to the HAPs and piretanide alone, and then to the hybrids. In Figure 1, the patterns of piretanide (A) and those of the HAPs and hybrids (B) are shown. 

Piretanide (Figure 1A) is a crystalline drug whose reflections agree well with those reported in the literature for Form A [16,17,19]. No impurities or traces of the other known polymorphic forms of piretanide were present. 

The doped HAPs as well as the hybrids with piretanide show over-imposable patterns (Figure 1B) in terms of both their intensities and peak positions. All the peaks agree well with the expected reflections of hydroxyapatite (see red bars in Figure 1B). No secondary phases are present. The peaks of piretanide are not evident, maybe due to its small amount (see the next sections) or to its conversion to an amorphous phase, as also occurred for other drugs in analogous hybrids [24,25]. Patterns of the physical mixtures (prepared as explained in Section 2.2.2) were also collected (Figure S2). Some deviations from the background level corresponding to the drug peaks can suggest its presence in the physical mixtures, even if their location in the high bump at a low angle and the low amount of drug make it difficult to confirm this observation. So, XRPD is not able to identify the possible presence of the drug in the hybrids and its nature (crystalline or amorphous). 

**Figure 2:**
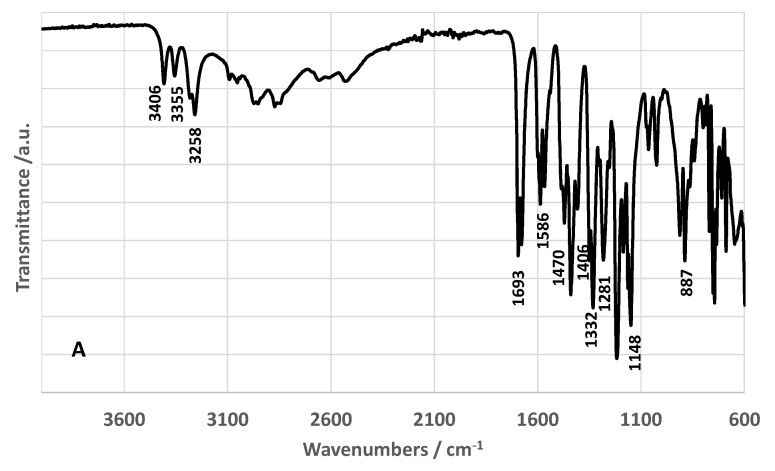

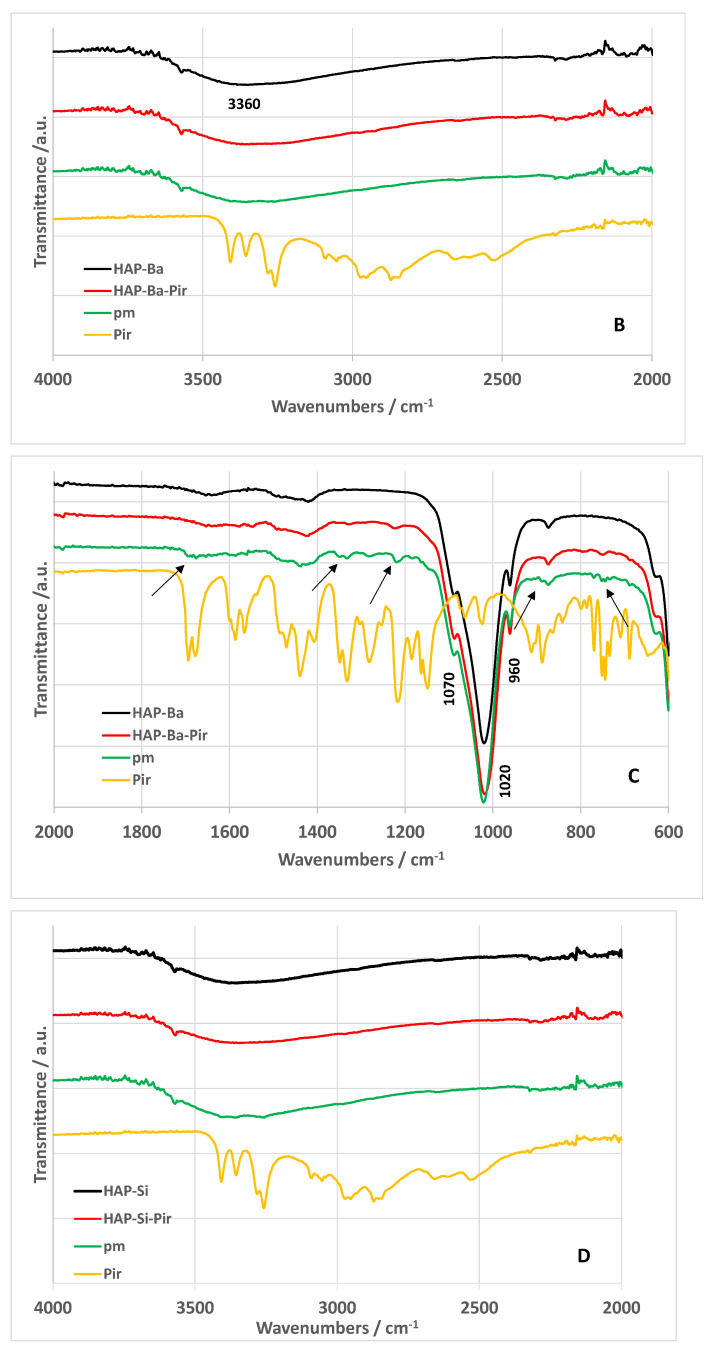

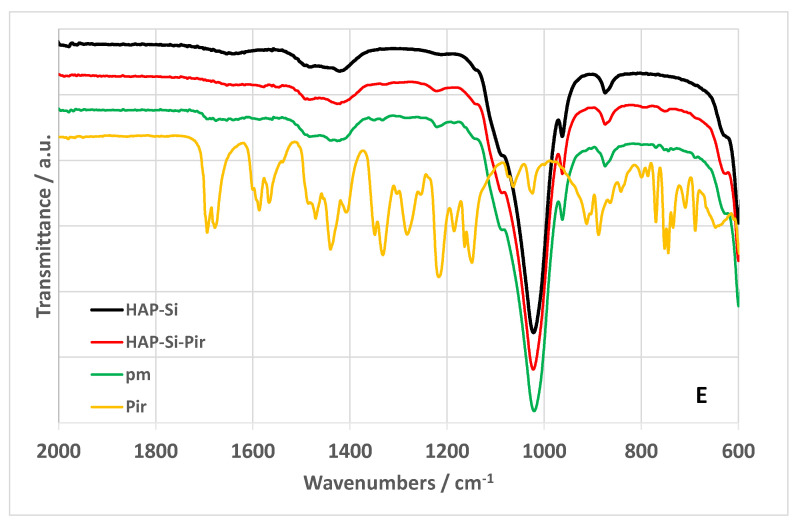
FT-IR spectra of piretanide (**A**) and the HAPs, hybrids, and physical mixtures in the 4000–2000 cm^−1^ and 2000–600 cm^−1^ spectral ranges for Ba doping (**B**,**C**) and Si doping (**D**,**E**), respectively. The arrows in (**C**) mark the main drug peaks in the physical mixtures and the hybrids (also valid for image (**E**)).

The piretanide adsorption onto the HAP nanoparticles could be investigated by means of spectroscopic analysis. The FT-IR spectrum of Pir is reported in Figure 2A, while those of the HAPs, hybrids, and physical mixtures are compared in the 4000–2000 cm^−1^ and 2000–600 cm^−1^ spectral ranges in Figure 2B–E. 

The pure piretanide drug spectrum (Figure 2A) agrees well with that reported in the literature [17,19] and is attributed to the polymorphic Form A, as also determined by XRPD analysis. The drug has some main absorptions located at 3406 cm^−1^ (OH stretching of carboxylic groups), 3355 and 3258 cm^−1^ (NH_2_ stretching of sulfonic groups), 1586–1470–1406 cm^−1^ (aromatic ring stretching), 1693 cm^−1^ (C=O stretching), 1281 and 887 cm^−1^ (asymmetric and symmetric stretching of aromatic C–O ethers), and 1332 and 1148 cm^−1^ (SO_2_ asymmetric and symmetric stretching).

The FT-IR spectra of the two HAPs are similar (Figure 2B–E), which are in good agreement with those reported in the literature for hydroxyapatites [24,26]. Only a few bands are evident in the medium infrared spectral range: the very broad band at about 3360 cm^−1^ is typical of the OH stretching mode, and the bands at 1070 and 1020 cm^−1^ and at 960 cm^−1^ are attributed to P-O antisymmetric and symmetric stretching modes, respectively.

In the hybrids’ FT-IR spectra, in addition to the bands previously commented on for HAP, some small peaks due to the drug are visible, particularly in the range 2000–600 cm^−1^ (see arrows in Figure 2C). Their positions, if compared with the positions of the drug alone, seem slightly shifted toward lower wavenumbers, suggesting the presence of electrostatic interactions between the inorganic matrix and the drug. These peaks demonstrate the presence of the drug in the hybrids, probably in small amounts due to the peak intensities. Similar spectra are observed for the physical mixtures; the drug peaks are more evident, but the shifts seem less evident. These observations can support the hypothesis of an interaction between the drug and HAP in the hybrids [27], which, in contrast, seems to be absent in the physical mixtures. 

**Figure 3:**
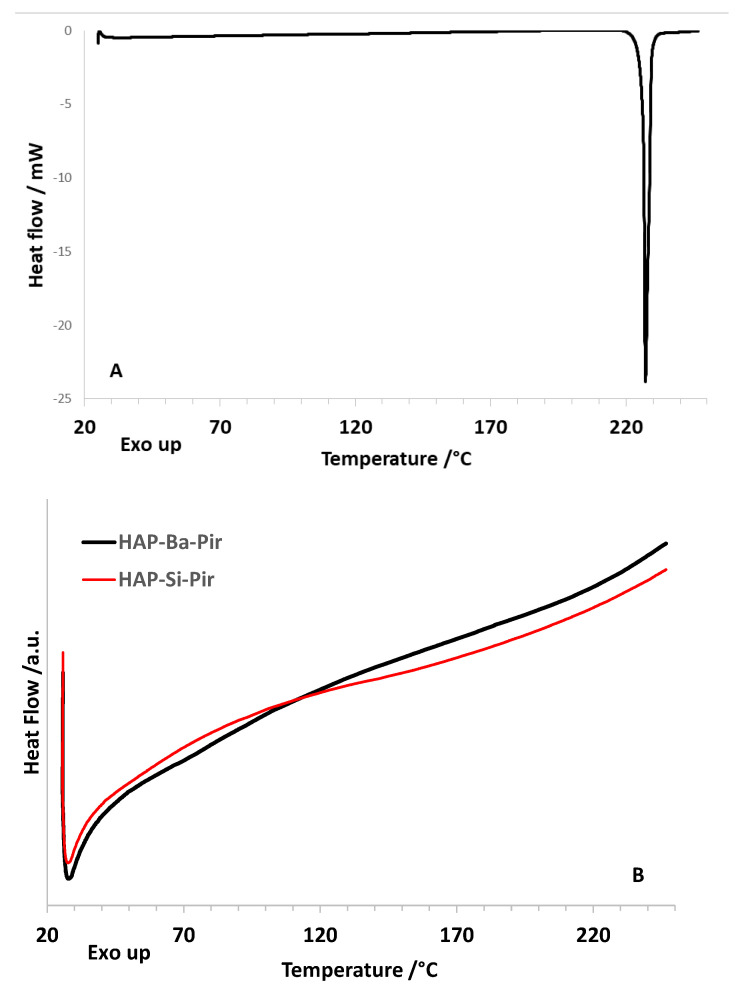
DSC curves of the Pir (**A**) and HAP-Ba-Pir and HAP-Si-Pir (**B**) samples.

The observation of melting or decomposition peaks of drugs in the calorimetric curves is particularly useful to prove drug adsorption onto inorganic matrices. The DSC curves of Pir and the hybrids are reported in Figure 3A,B, while those of the physical mixtures are reported in Figure S3.

For Pir (Figure 3A), a good agreement with the literature is found [19]. The only thermal event in the analyzed temperature range is the melting process at about 226.4 °C (ΔH = 116.7 J/g), which confirms the polymorphic Form A for piretanide [16,17]. No peaks due to polymorphic transition or drug decomposition can be seen.

Both the hybrids have similar DSC traces (Figure 3B). It should be underlined that HAP is highly stable in the analyzed temperature range, as are other analogous inorganic compounds [28]. The curves in Figure 3B are flat and without thermal events, apart from a broad band due to water desorption at low temperature, proving that piretanide turned out to be amorphous in the hybrids because of its adsorption onto the HAP surfaces. It is possible that the structural disorder introduced in the HAPs due to the presence of the dopant, which is more marked on the surfaces, and the nanometric dimensions of HAP particles could favor the amorphization of piretanide onto HAP nanoparticles, which would be a successful result because the drug can be more bioavailable with respect to its crystalline form. In addition, the low degree of crystallinity of the hybrids could be considered a positive aspect of the obtained DDS, possibly favoring its dissolution rate. 

By inspecting the DSC curves of the physical mixtures (Figure S3), the melting peak of the drug is evident at the same temperature as the pure drug (Figure 3A). This evidence clearly proves that the drug is in an amorphous state in the hybrids.

**Figure 4:**
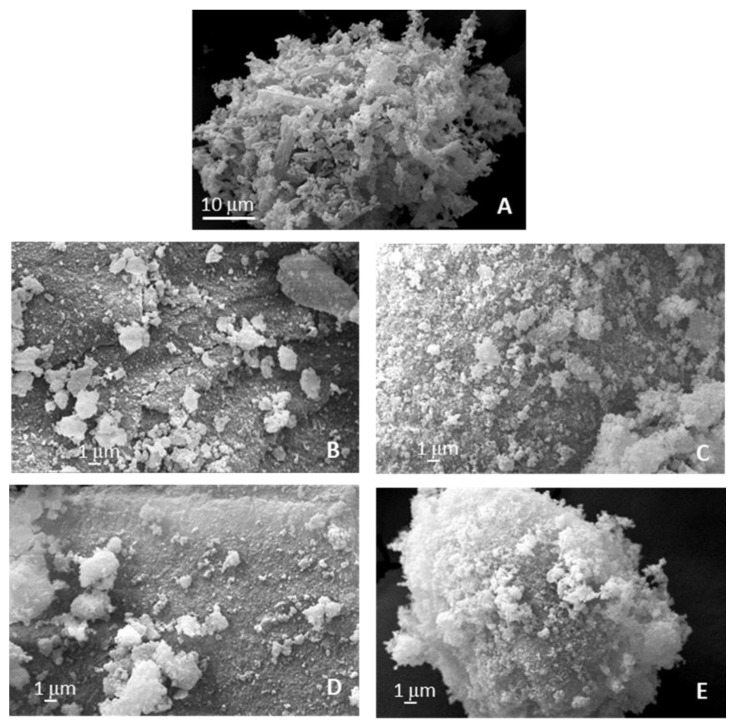
SEM images of the (**A**) Pir, (**B**) HAP-Ba, (**C**) HAP-Si, (**D**) HAP-Ba-Pir, and (**E**) HAP-Si-Pir samples. The magnifications were 5000 kX and 10,000 kX for the (**A**) and (**B**–**E**) images, respectively.

The morphological characterization was performed by SEM analysis. The micrographs of Pir, the HAPs, and the hybrids are shown in Figure 4A–E. 

Piretanide appears in the form of rod-shaped crystal aggregates, with a length and width of several microns (Figure 4A). In contrast, HAP-Ba is comprised of compact aggregates of small, rounded particles (Figure 4B), and the same is true for the corresponding hybrid (Figure 4D). The HAP-Si sample is also composed of aggregates of rounded particles (Figure 4C) that, however, maintain their identity and seem more separated with respect to the Ba-doped sample. The hybrid (Figure 4E) is again very similar to the corresponding HAP. Typical drug particles cannot be seen. Similar morphologies were reported for analogous hybrids based on HAPs [24,25,29].

A widespread tool to check the stoichiometry of the samples is EDS microanalysis, which, when combined with SEM, can support successful hybrid formation by analyzing the presence of the characteristic elements of HAP (Ca and P ions and the substituents Ba and Si) and piretanide (S ions). For the different samples, from their stoichiometry (see Section 2.1), the cationic ratio can be calculated as Ba/Ca = 0.053, Ca/P (Ba doping) = 1.58, Si/P = 0.071, and Ca/P (Si doping) = 1.78. For all the samples, the EDS analysis (see, for example, the HAP-Si-Pir spectrum in Figure S4) allowed us to find the expected elements and the chemical compositions (Table 1). 

The Ca/P ratios are in quite good agreement with the expected values, while the dopant amount is, in both cases, about half of the expected values. This can be due to loss of the dopant during the synthesis in solution but also to a solubility limit for the dopant ions in the HAP structure. The presence of the S peak, the element peculiar to piretanide, is evidence confirming the presence of the drug in the analyzed samples. From the atomic ratios (Table 1), we can calculate the drug loading. The drug loading (wt%) can be expressed as MW_Pir EDS_/(MW_HAP_ + MW_Pir EDS_). So, starting from the values reported in Table 1 and the molecular weight of Pir (362.4g/mol), HAP-Ba (1052 g/mol), and HAP-Si (1003 g/mol), we can determine the values of 362.4 × 12/(362.4 × 0.12 + 1052) = 0.04 and 362.4 × 0.2/(362.4 × 0.2 + 1003) = 0.067 for HAP-Ba-Pir and HAP-Si-Pir, respectively. These data will be compared with the drug loading amount determined by UV-Vis spectroscopy (see Section 3.2). 

### 3.2. Pharmaceutical Characterization

As described in the experimental part (Section 2.3.2), we can calculate the drug loading from UV-Vis measurements. These were rather low: 2.9 ± 0.4% for HAP-Ba-Pir and 3.6 ± 0.6% for HAP-Si-Pir, respectively. These values can be compared with those obtained from EDS measurements and are found to be in quite good agreement, within the standard errors of each kind of measurement. In particular, both measures suggested that Si-doped HAP can adsorb a slightly higher amount of drug with respect to the barium-doped HAP, probably due to its morphological features. The solubility of the hybrids was significantly increased compared with the drug alone in the two fluids considered (Table 2). In both cases, it was so high that we had to stop the experiment because too much sample was needed to saturate the solutions.

**Figure 5:**
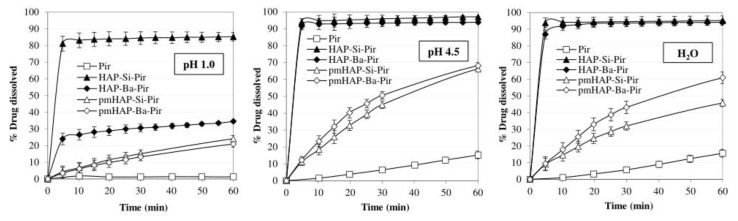
Dissolution profiles of piretanide alone compared with those of the HAP-Ba-Pir and HAP-Si-Pir hybrids and the physical mixtures, pmHAP-Ba-Pir and pmHAP-Si-Pir, in the three fluids considered. The dose was always 6 mg.

The dissolution rate of the drug from the two proposed hybrids was quite fast in all three fluids considered (Figure 5), especially for HAP-Si-Pir. In fact, at pH = 1, the condition simulating administration on an empty stomach and in which the drug is less soluble, the dissolution of HAP-Si-Pir reached approximately 85% of the dose in 5 min and that of HAP-Ba-Pir reached about 30% of the dose, while only 3% of the dose of pure piretanide passed into the solution in one hour. Even the two physical mixtures showed very slow dissolution rates, demonstrating that hybrid formation is crucial for the efficacy of the delivery system. 

In the other two fluids, the solubilization of the dose was complete, i.e., 100% of the dose was dissolved, within 5 min for both HAP-Si-Pir and HAP-Ba-Pir. The physical mixtures, pmHAP-Si-Pir and pmHAP-Ba-Pir, showed some increase in the dissolution rate of the drug, but only the hybrid compounds could guarantee passage into solution of piretanide in such a short time, and this factor is extremely important in making the drug available for absorption. The Si-doped hybrid seemed to be the best system for a satisfactory release of piretanide in all the tested fluids [30].

To highlight the similarity or difference between the different dissolution profiles, the f_2_ similarity test was used, and the results are reported in Table S1.

**Figure 6:**
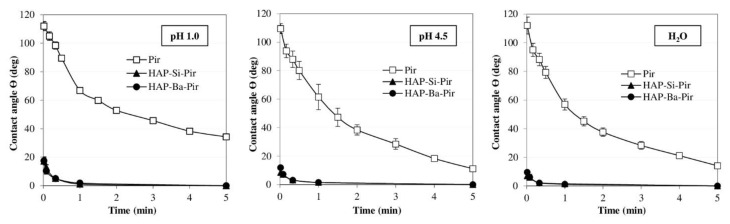
Contact angle θ of piretanide alone compared with that of the HAP-Ba-Pir and HAP-Si-Pir hybrids in the three fluids considered.

Contact-angle measurements can further explain the causes of the remarkable improvement in the dissolution behavior of the proposed samples in all three media considered (Figure 6) [31]. In fact, the contact-angle values demonstrate the hydrophobicity and low wettability of piretanide. In contrast, the hybrid compounds show much greater hydrophilicity: in both cases, the contact angle drops to zero in a few seconds. 

## 4. Conclusions

The successful loading of piretanide onto doped HAP was proved with the combined use of different experimental techniques; in particular, by spectroscopy and electronic microanalysis. The drug, loaded onto HAP surfaces, turned out to be amorphous, which is an amazing result because this means that it could be more bioavailable compared with the crystalline form. In addition, the low crystallinity of the hybrid could also help to improve the dissolution rate. Pharmaceutical measurements demonstrated rapid drug release in different fluids simulating the gastrointestinal conditions after oral administration. In particular, at pH = 1, in which the drug is less soluble, HAP-Si-Pir released 85% of the dose and HAP-Ba-Pir 30% of the dose (compared with 3% in 1 h for the drug alone), while in all the other tested conditions, the entire dose was released in about 5 minutes for both hybrids (the drug alone can reach about 10% in 1 h). This remarkable improvement in the dissolution rate can be explained by an increase in the wettability and solubility of the drug loaded in the drug-carrier systems.

The adsorption of piretanide onto the HAP surface was a promising strategy, notwithstanding that the drug loading was fairly low. To increase the loading and to obtain highly performing systems based on HAP hosts, the optimization of HAP, in particular its morphology and/or composition, could be developed to extend this strategy in the future to other poorly soluble drugs.

## Figures and Tables

**Table 1:** Atomic percentages of the elements obtained by EDS analysis. The values are the average of the measurements at three different sample points.

Samples	Ca (atomic %)	P (atomic %)	Dopant (atomic %)	S (atomic %)	Ca/P	Dopant/Ca or Dopant/P
HAP-Ba	16.21	11.47	0.36		1.41	0.023
HAP-Si	12.02	7.83	0.14		1.53	0.02
HAP-Ba-Pir	14.61	10.08	0.47	0.12	1.45	0.032
HAP-Si-Pir	10.66	7.33	0.2	0.2	1.45	0.027

**Table 2:** Solubility of the hybrids in comparison with the drug alone.

Equilibrium Solubility at 21 °C (mg/L)		
Samples	Deionized Water	HCl 0.1 N (pH 1.0)
Piretanide	35.1 ± 3.2	21.1 ± 0.7
HAP-Si-Pir	>155	>60.9
HAP-Ba-Pir	>137	>68.7

## Data Availability

Data available on request.

## Supplementary Materials

The following supporting information can be downloaded at: https://www.mdpi.com/article/10.3390/pharmaceutics16111450/s1, Figure S1: Ionization percentage of the drug piretanide at the different pHs considered. Figure S2: XRPD patterns of the physical mixtures compared with that of piretanide. Figure S3: DSC curves of the physical mixtures pmHAP-Ba-Pir (A) and pmHAP-Si-Pir (B). Figure S4: EDS spectrum of HAP-Si-Pir. Table S1: Similarity test f_2_ of the dissolution profiles reported in Figure 5.
