# Supplementary material for: A Perspective on the Use of Hydroxyapatites to Improve the Dissolution Behavior of Poorly Water-Soluble Piretanide
Source: Pharmaceutics. 2024 Nov 13;16(11):1450. doi: 10.3390/pharmaceutics16111450 (PMC11597431; doi:10.3390/pharmaceutics16111450)
Supplement: Supplementary file 1 [file pharmaceutics-16-01450-s001.zip › pharmaceutics-3301987-supplementary.pdf]

pharmaceutics

Article

Supplementary Materials: A Perspective on the Use of Hydroxyapatites to Improve the Dissolution Behavior of Poorly Water-Soluble Piretanide

Valeria Friuli, Claudia Loi, Giovanna Bruni, Lauretta Maggi and Marcella Bini

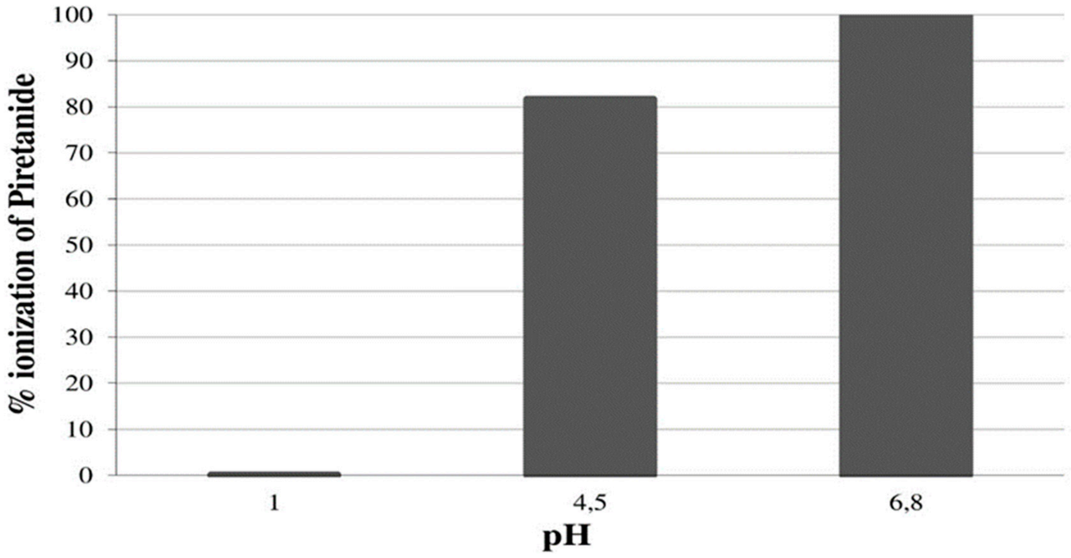

Figure S1. Ionization percentage of the drug piretanide, at the different pH considered.

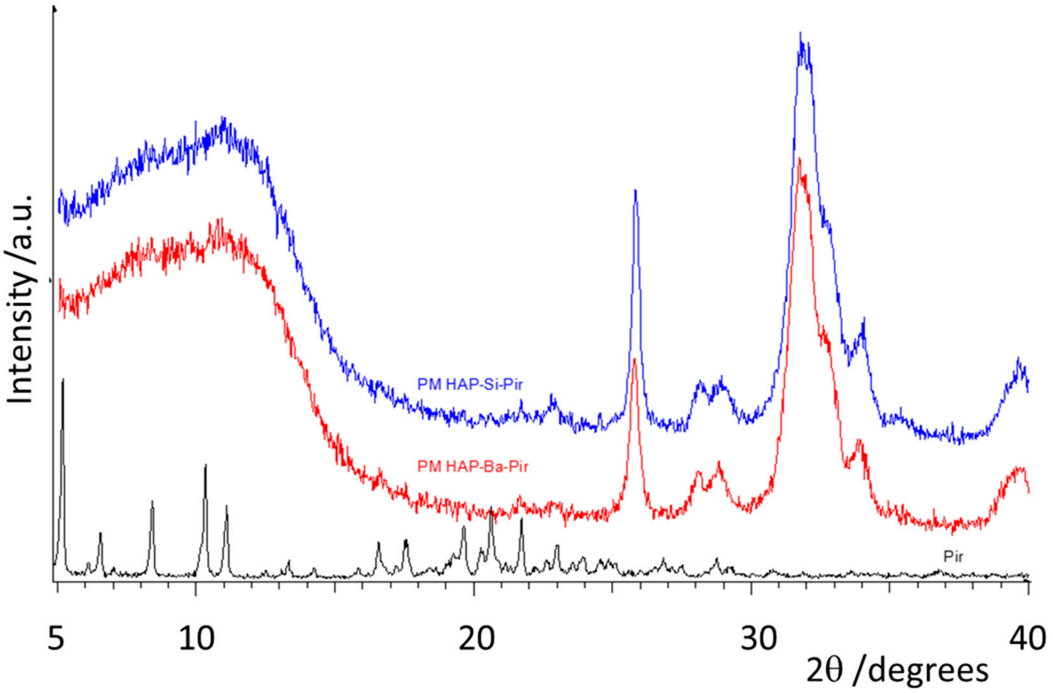

Figure S2 XRPD patterns of physical mixtures, compared with that of piretanide.

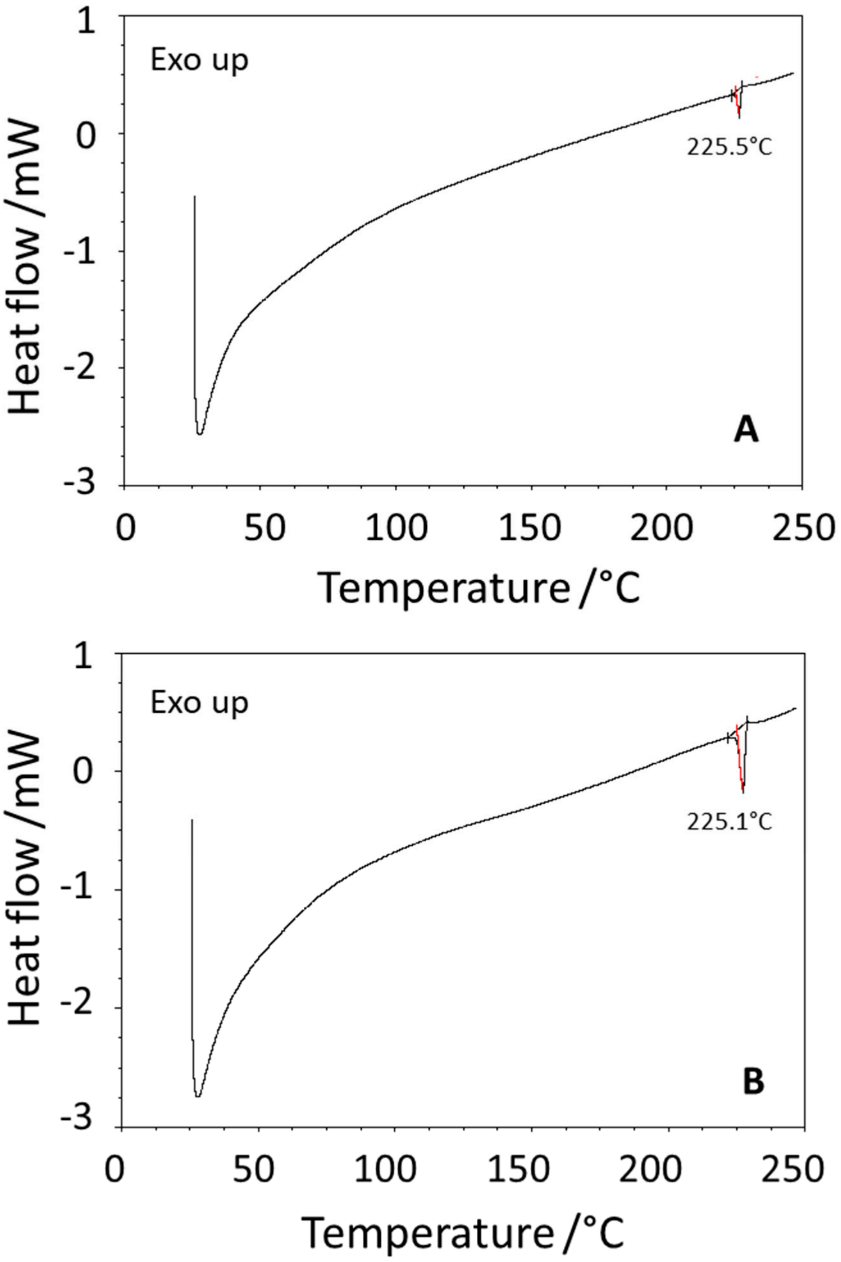

Figure S3. DSC curves of physical mixtures pmHAP-Ba-Pir (A) and pmHAP-Si-Pir (B). The red line marks the onset temperature extrapolation.

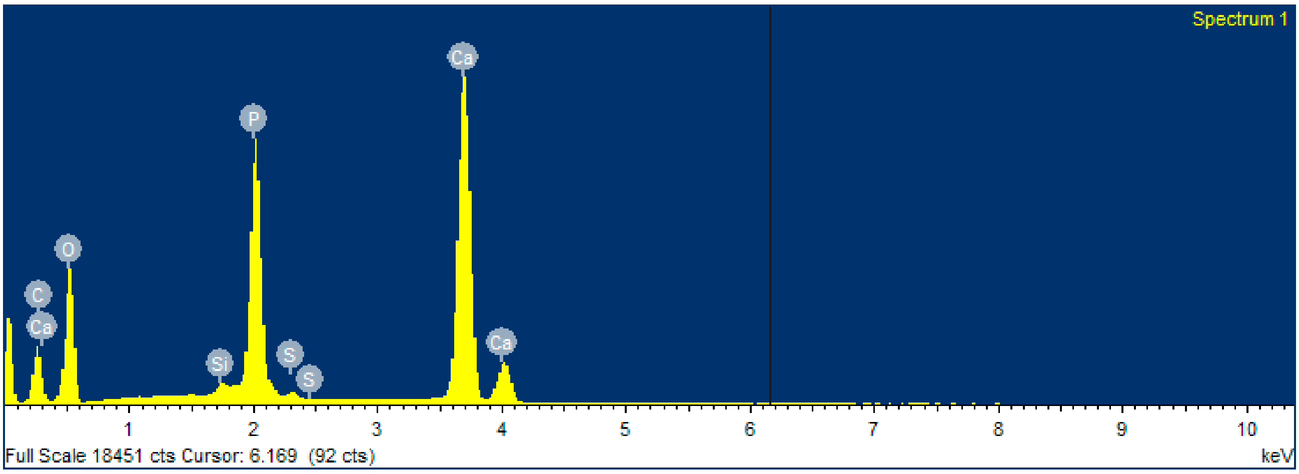

Figure S4. EDS spectrum of HAP-Si-Pir. The circles mark the peaks due to the elements found in the sample.

Table S1. Similarity test f_2 of the dissolution profiles reported in figure 5 (see text).

	**Similarity factor (f_2)**		
**Samples**	**pH 1.0**	**pH 4.5**	**pH 6.8**
Pir versus HAP-Si-Pir	NS (4.0)	NS (2.8)	NS (3.0)
Pir versus HAP-Ba-Pir	NS (26.4)	NS (4.6)	NS (3.3)
pmHAP-Si-Pir versus pmHAP-Ba-Pir	S (84.4)	S (66.1)	NS (7.6)
HAP-Si-Pir versus pmHAP-Si-Pir	NS (7.7)	NS (9.9)	NS (45.7)
HAP-Ba-Pir versus pmHAP-Ba-Pir	NS (36.5)	NS (14.2)	NS (10.6)
HAP-Si-Pir versus HAP-Ba-Pir	NS (13.4)	S (56.8)	S (80.2)

S = Similar.

NS = Not Similar.
